# A practical approach to the ethical use of memory modulating technologies

**DOI:** 10.1186/s12910-020-00532-z

**Published:** 2020-09-18

**Authors:** Shawn Zheng Kai Tan, Lee Wei Lim

**Affiliations:** School of Biomedical Sciences, Li Ka Shing Faculty of Medicine, The University of Hong Kong, 21 Sassoon Road, Pokfulam, Hong Kong, PR China

**Keywords:** Memory, Neuroscience, Technology, Golden mean, Authenticity

## Abstract

**Background:**

Recent advancements in neuroscientific techniques have allowed us to make huge progress in our understanding of memories, and in turn has paved the way for new memory modification technologies (MMTs) that can modulate memories with a degree of precision, which was not previously possible. With advancements in such techniques, new and critical ethical questions have emerged. Understanding and framing these ethical questions within the current philosophical theories is crucial in order to systematically examine them as we translate these techniques to the clinic.

**Main body:**

In this paper, we discuss the ethical implications of modern neuroscience techniques that aim to disrupt or enhance memories. We attempt to frame the MMTs in the context of existing ethical philosophical theories to provide a cohesive analysis of the myriad of ethical quagmires that might emerge from such technologies. We argue the application of Aristotle’s Golden Mean and multiple accounts of authenticity are useful in approaching the ethical questions surrounding MMTs. We then propose a framework in which ethical considerations can be systematically examined. Lastly, we provide caveats and considerations for the use of this framework. Overall, we provide a practical approach for the ethical use of MMTs depending on the situation.

**Conclusion:**

While at face value, our model appears to put severe limitations on the application of MMTs, we are not completely opposed to their use, but rather our framework guides the agent to consider the implications before making any decisions. Most importantly, we argue that the use of MMTs does not reduce the responsibility of the initial decision, and the agent must accept the post-MMT self as the new “true self” regardless of the outcome. As the developmental trajectory of MMTs suggests we are getting closer to practical clinical applications, ethical concerns across a wide range of disciplines need to be addressed to develop best strategies and policies when dealing with MMTs. If this can be achieved, we believe the ethical use of MMTs is not only possible but would also be of tremendous benefit to many people suffering from memory-related mental disorders.

## Background

With successive advancements in the neuroscience of memories, we are slowly but surely beginning to understand how to modulate memories. The ability to manipulate memory processes such as creating false memories [1, 2], erasing or silencing memories [3, 4], and altering emotional valences of memories [5] has already been achieved in animal models and brings us a step closer to human applications. While pursuing excellence through intellect by rigorous scientific experiments is indeed crucial, it is important that we also consider the ethical implications of such powerful technologies [6, 7]. Quoting the seminal works of Aristotle in Nicomachean Ethics [8]:

“The Excellence of Man then is divided in accordance with this difference: we make two classes, calling the one Intellectual, and the other Moral” (Nicomachean Ethics, Book 1, Chapter XIII).

“Surely then, even with reference to actual life and conduct, the knowledge of it [Chief Good] must have great weight; and like archers, with a mark in view, we shall be more likely to hit upon what is right: and if so, we ought to try to describe, in outline at least, what it is and of which of the sciences and faculties it is the End.” (Nicomachean Ethics, Book 1, Chapter II).

Given the aim of much of the research on Memory Modulating Technologies (MMTs) is the translation to clinical applications, then surely the ethical considerations of using such technologies that can affect the happiness/welfare of an individual and society (eudaimonia) would be necessary for the “Chief Good”.

In this paper, we therefore attempt to develop a framework based on existing ethical theories that systematically examines the ethical consequences of the use of MMTs, as well as provide caveats and considerations for the use of this framework. Overall, we provide a practical approach to how MMTs, such as DBS, could be ethically used in certain situations.

## Main text

This paper consists of seven sections. In section 1, we discuss the current advancements in MMTs and briefly describe the current state of both memory enhancing and memory editing technologies, including the general science behind them, as well as their capabilities and limitations. In section 2, we describe Aristotle’s Golden Mean and briefly contextualize it in terms of memories and MMT. In section 3, we describe three forms of authenticity described by Erler [9] and similarly contextualize them in terms of memories and MMT. In section 4, we describe the ethical challenges of MMTs in terms of both the Golden Mean and authenticity, as well as give some examples. In section 5, we introduce a practical model that aims to systematically use the above theoretical frameworks. In section 6, we discuss some caveats and considerations on the use of this model and then examine how they can be approached. Lastly, in section 7, we conclude the paper with some thoughts on the ethical use of MMTs. Overall, our model based on ethical philosophical theories allows physicians and patients to systematically assess the appropriateness of the use of MMT. We conclude that the use of MMT does not reduce the responsibility of the initial decision, and the agent choosing to use MMTs (assuming that they are informed to a reasonable degree) must accept the post-MMT self as the new “true self” regardless of the outcome.

### Memory modification technologies

Before discussing the philosophical underpinnings of the use of MMTs, it would be rational to highlight some of the progress in MMTs for a clearer understanding of the current state and limitations of such technologies, and future advancements. This will be a brief overview of MMTs, as this topic has been recently covered by multiple groups [6, 7, 10]. According to Liao and Sandberg [11], MMTs can be broadly categorized into two groups: memory enhancing and memory editing technologies, both of which have already been discussed by us in relation to neuromodulation [6, 10].

#### Memory enhancement

Memory enhancement MMTs are interventions that are designed to improve certain aspects of memories. Memory enhancement can be simply considered to be the improvement of memories above that of normal functioning and are often thought to be separate from therapeutics that restore normal memory function. However, in this paper, we will consider memory enhancement MMTs as encompassing both improvement and restoration.

Memory enhancement MMTs have been in existence for a long time, for example, humans have long been using techniques such as mnemonic devices to enhance memory processes. However, with modern advancements in neuroscience and our increased understanding of the brain, we are starting to see the emergence of drugs and devices that can enhance memory by directly manipulating neurological functioning. For example, the use of d-cycloserine, an N-methyl-D-aspartate (NMDA) partial agonist, has been shown to facilitate memory consolidation [12–14]. Similarly, neuromodulation techniques like Deep Brain Stimulation (DBS) have been shown to enhance memory functions in rodents using spatial learning as a proxy for hippocampal learning. The hippocampus is a common target for memory enhancement by MMTs for treating dementias like Alzheimer’s disease, as the hippocampus is one of the first areas to exhibit atrophy [15, 16]. Techniques such as DBS could be beneficial for Alzheimer’s disease in the preclinical stages, and such treatments are being rapidly developed and translated to humans, and multiple clinical trials have already been done [17–22]. However, it should be noted that the use of DBS is not without its limitations. Phase II trials of DBS for treating Alzheimer’s disease have had limited success and effects were only seen in patients 65 years old and above. Although there was a slowing of cognitive decline over 1 year, this appeared to be un-sustained at 12 months and memory still worsened overall [21]. This same study also showed that DBS applied to patients below 65 years of age might actually worsen their memory [21]. Other studies showed that DBS had paradoxical memory impairment in certain scenarios [23–25]. Non-invasive neuromodulation techniques that can alter memories have also been investigated, however, DBS currently has the advantage of higher spatial and temporal specificity and hence more controllable [6]. Modern non-invasive neuromodulation technologies, like focused ultrasound DBS, are however starting to challenge this notion, and have been shown to be able to stimulate or suppress neural activity with high spatial accuracy, allowing for modulation of neuronal circuits in a non-invasive manner [26–29]. Regardless, non-invasive neuromodulation techniques have faced similar significant challenges in their development [30]. Even though all these techniques are incredibly limited and their precise mechanisms of action are unknown, they do show potential in enhancing memories beyond natural enhancement methods such as mnemonic devices. Modern memory-enhancing technologies including pharmacological or device-based methods generally aim to enhance synaptic plasticity, a widely accepted putative neural mechanism for learning and memory in which the strength of synaptic transmission is either upregulated or downregulated [31, 32]. This fundamental concept was first described by Hebb [33], in which synaptic changes were observed when a cell excites another cell repeatedly or is consistently involved in the excitability. Such processes cause synaptic changes that increase the efficiency of the first cell firing the second cell. Some modern memory enhancing techniques focus on manipulating synaptic plasticity in the brain to enhance memory processes by enhancing neurogenesis or synaptogenesis, by modulating the transmission of memory-related neurotransmitters and their receptors, or by disinhibiting neuronal activity involved in the memory process. However, memory enhancement MMTs face challenges in their specificity, as increasing synaptic plasticity could enhance “off-target memories”. A possible mechanism is Long-TermPotentiation (LTP), the most studied form of synaptic plasticity, in which long-term enhancement in synaptic excitability results from the coincident activity of pre- and post-synaptic elements [34]. Memory enhancement MMTs could be designed to increase LTP efficiency to increase memory function. However, LTP is found in all excitatory pathways in the hippocampus [35], so enhancing LTP would lead to an overgeneralized increase in learning and memory functions, which in turn could have severe implications for diseases such as addiction and anxiety disorders.

#### Memory editing

Memory editing MMTs are interventions that are designed to modulate existing memories including changing the valance of a memory, adding false memories, or even erasing memories. Similar to memory enhancement MMTs, memory editing MMTs can come in many forms including psychological methods, drugs, and devices. For example, extinction therapy (laboratory-based exposure therapy) during memory reconsolidation when memory is susceptible to change [3] has been shown to be effective in removing fear memories [36, 37]. Shaw and Porter [38] were able to use suggestive memory-retrieval techniques to induce false memories in 70% of the participants. Pharmacological memory editing MMTs have also been explored, of which the most well-known is propranolol, a β-adrenergic receptor antagonist. Administration of propranolol before or after memory retrieval was shown to disrupt memory reconsolidation leading to erased memories [39, 40]. Neuromodulation techniques such as Transcranial Magnetic Stimulation (TMS), Transcranial Direct Current Stimulation (tDCS), and DBS have been suggested to also erase fear memories (the mechanisms have been previously discussed in detail [6]); for example, DBS was able to disrupt consolidation of fear memories in a rodent model [41]. Optogenetic technologies have been used to directly manipulate specific fear memories in rodents to create false memories [1], change the valence of memories [42], and selectively activate and inactivate memories [4]. Recently, researchers have even used optogenetic stimulation to create de novo memory [2]. Although this technology currently requires transgenic animals and/or precise manipulations using viruses, which makes it unfeasible to translate to humans, it does open up the possibility that MMTs could eventually be developed with the ability to edit human memories. Memory editing MMTs also currently face issues of specificity, as it can be difficult to target correct memories, which in turn could exacerbate the problem [43, 44]. Furthermore, the aforementioned studies mainly focused on the action of MMTs on the hippocampus, a structure that plays a critical role in declarative memories. Although the use of rodent models can provide good translational value to humans, the inability to detect changes in declarative memory in animals risks unwanted possible negative side effects on declarative memory in humans. Furthermore, there are concerns over the efficacy and safety of these techniques, and the contradictory nature of the empirical studies [10, 45, 46]. Considering all these findings, there are many potential ethical concerns particularly the transition to clinical studies in humans.

### Golden mean

#### Introduction

In Aristotle’s Nicomachean Ethics [8], he argues that moral virtue is moderation, a relative mean between excess and deficiency, what we call the “Golden Mean”. He writes:

“So then it seems every one possessed of skill avoids excess and defect, but seeks for and chooses the mean, not the absolute but the relative.” (Nicomachean Ethics, Book 2, Chapter VI).

In this, he argues that to lead a virtuous and good life, one must strive for moderation. For example, courage is seen as a virtue, whereas a lack of fear can be seen as recklessness, and too little courage can be seen as cowardice. Similarly, generosity is a virtue, whereas too much can be seen as extravagance and too little can be seen as miserly. Overall, he argues that eudaimonia or a state where we are flourishing is achieved through the Golden Mean, in which we do not live in excess or in deficit. It should be noted that this mean is not fixed but rather relative depending on the circumstances of an individual. For example, excessive eating is considered gluttony, but the amount of food might be considered necessary depending on an individual’s needs, such as the different dietary requirements of an athlete versus an office worker.

#### Golden mean, memory, and emotions

How then does this Golden Mean relate to memory and MMTs? The most direct answer would be that we should strive towards a Golden Mean for memory—we do not remember too much or too little. Although this seems like a perfect argument for memory enhancement MMTs to restore memory deficits in disorders like dementia by restoring memory function, it becomes a little bit more complex when considering memory editing MMTs. For example, vmPFC DBS could be used to disrupt fear memories [6] by lowering memory function with the aim to achieve the Golden Mean for memories, but the overall effect is on a single memory and the functionality of the overall memory system remains intact. Aristotle writes [8]:

“It is possible, for instance, to feel the emotions of fear, confidence, lust, anger, compassion, and pleasure and pain generally, too much or too little, and in either case wrongly; but to feel them when we ought, on what occasions, towards whom, why, and as, we should do, is the mean, or in other words the best state, and this is the property of Virtue.” (Nicomachean Ethics, Book 2, Chapter VI).

In this case, we argue that memory editing MMTs can help target emotional memory and restore balance to emotions. For example, a soldier may suffer from post-traumatic stress disorder (PTSD) associated with loud noises of gunshots that might be an appropriate fear response in a warzone. However, on returning to a city from the warzone, the fear response to normal city sounds might be maladaptive and could be seen as inappropriate in this context. Using memory editing MMTs to disrupt memory could then be seen as achieving the Golden Mean by limiting the excessive fear response towards loud noises. The difficulty in this scenario, as mentioned above, is that specificity and accuracy remain a major issue.

However, within the context of achieving the Golden mean, other issues of MMTs emerge. Although memory enhancement MMTs might be able to restore memory in patients with dementia, an overgeneralized increase in memory might lead to the persistence of unwanted memories. For example, a person treated with a memory enhancement MMT might be unable to forget minor personal failures due to the stronger imprinting of negative emotional implicit memories associated with that event, which in turn might lead to lower self-esteem and higher levels of anxiety. In this case, the memory enhancement MMT might achieve the goal of improving memory, but the harmful emotional side effects from unwanted memories would mean a failure to achieve the Golden Mean. A possible scenario is that the agent recognizes their personal failures as minor and understands this response is caused by the MMT, but would this still create high levels of anxiety? We argue that anxiety is a disease of irrationality, in that regardless of the rational knowledge, the implicit memories (such as Pavlovian conditioning) would still trigger physiological responses. Therefore, regardless of the knowledge of the negative side effects of MMTs and the rational thought processes relating to these negative side effects, the physiological responses would still cause issues such as anxiety. Similarly, while disrupting memories using memory editing MMTs might be able to lower maladaptive emotions, it could also remove appropriate emotions. For example, an appropriate amount of guilt is a fitting response to certain wrongdoings, however, using memory editing MMTs to remove these emotions would not achieve the Golden Mean as the individual would no longer “feel them [emotions] when we ought” and can easily fall into the vice of irresponsibility.

Overall, the nature of MMTs has many facets that can inevitably result in many unwanted effects. Although MMTs might be perceived to fulfil their purpose, their effects on other facets might result in the agent being further away from the Golden Mean. These issues are complex and will be further discussed in section 4 which looks at specific ethical issues of MMTs. Overall, using Aristotlean ethics as a framework results in arguments on both sides for the ethical permissibility of using MMTs to alter memories.

### Authenticity

#### Introduction

One issue that has been raised in the objection to the use of MMTs is the threat to human authenticity [9, 47]. Specifically, the concern is that MMTs allow us to control and manipulate our memories, which in turn would lead to situations where we would be unable to be “true” to ourselves, or inauthentic. Quoting from the report Beyond Therapy by the President’s Council on Bioethics [47]:

“But if enfeebled memory can cripple identity, selectively altered memory can distort it. Changing the content of our memories or altering their emotional tonalities, however desirable to alleviate guilty or painful consciousness, could subtly reshape who we are, at least to ourselves.” [p210].

“More precisely, we might come to pursue such happiness (through manipulating memories) by willingly abandoning or compromising our own truthful identities.” [p227].

The report states that altering memories can “reshape who we are”, and in turn, compromise our own truthful identity, which can be interpreted as a threat to authenticity. However, before discussing these implications, it would be useful for us to briefly define authenticity. Here, we use the three definitions of authenticity described by Erler [9], namely wholeheartedness, the existentialist’s account of authenticity, and “true self”. Attributed to Harry Frankfurt, wholehearted authenticity refers to that of an agent acting upon their preferences that they wholeheartedly believe and identify with. This means that their decisions are not made out of ambivalence, but rather based on their higher-order desires. The existentialist’s account of authenticity is similar to the concept of wholeheartedness, however, the conditions are more demanding. According to existentialists like Jean-Paul Sartre, authenticity is about making choices with autonomy by taking full responsibility and avoiding “*mauvaise foi”* or “bad faith”, a phenomenon in which an agent adopts false values due to external or social pressures and in doing so denies their freedom. In this aspect, the existentialist account of authenticity encompasses that of wholeheartedness (in which an authentic agent acts on their preferences and identity), but also includes an additional requirement that it is the agent’s own honest choice and does not involve pressure from social forces that causes one to disowns one’s freedom. Lastly, the “true self” account of authenticity refers to a narrative understanding of the self and central features, which takes into consideration the “frameworks” in which we live in (e.g., what is considered a successful life or what is meaningful), making it something that is in part “given” to us rather than fully self-constructed. Erler [9] writes that it is “the virtue of being faithful to one’s “true self” when doing so is intrinsically valuable”. This means that regardless of a person’s preference of identity, there are features of a person that count as their identity and hence their “true self”, which refers to the person’s real features and not ideal ones. It should be noted that “true self” is not a pre-given set of beliefs, feelings, and opinions, but rather a narrative construct of oneself that is affected by the frameworks by which we live in. For simplicity, we shall consider authenticity in the “true self” accounts to also include “clear-headedness” or “truthful living”, in which the agents have a reasonably accurate awareness of their narrative, and both past and present circumstances, which in turn shapes who they are. Although this has implications on what we consider to be self-deception (do we consider individuals who are honestly mistaken about themselves, and thus misrepresent themselves as self-deceiving?), for the purpose of this paper, these considerations have major implications only on the terminology rather than on actual arguments. We also note that there is a difference between “clear-headedness” and “true self”—one who is not clear-headed might live authentically in the “true self” accounts if they sincerely believe in an untrue narrative of their life. However, for the sake of simplicity, we shall take the “true self” accounts to also refer to clear-headedness, as the arguments in this paper tend to apply to both when speaking about “true self”. Although these three accounts of authenticity may differ in how they interpret what it is to live an authentic life, it should be noted that they are not necessarily contradictory, but rather complementary in many cases.

#### MMTs as a threat to authenticity

As mentioned before, a major concern of the use of MMTs is the threat to authenticity. But how do they affect authenticity in the context of the above accounts of authenticity? In terms of wholeheartedness, we argue that MMTs have the ability to alter our beliefs, and hence, what we identify with. Memory editing MMTs can possibly erase memories that shape our beliefs. For example, erasing childhood fear memories could alter our cautiousness that these experiences might have instilled in us. Similarly, memory enhancement MMTs can lead to overgeneralized enhancement of memory, and as mentioned above, could lead to remembering forgotten minor personal failures that might have a huge impact on one’s beliefs (i.e., changing risk-taking behaviour due to remembering one’s past failures). Would it then be possible to act wholeheartedly according to one’s beliefs and preferences if such beliefs and preferences might be altered by using MMTs? The existentialist account of authenticity adds further complexity to this by questioning if the use of MMTs would be considered to be in bad faith. Would the change of values caused by MMTs be considered as false values? To what extent is the use of MMTs due to societal pressures that in turn alter our values and decisions? Would the blame be placed on MMTs for one’s subsequent decisions and is this a form of disowning one’s freedom? These questions highlight the complexity of how MMTs might affect the existentialist account of authenticity. Lastly, in the “true self” account, the ability of memory editing MMTs to alter one’s past by changing one’s memories could alter the “true self” through the manipulation of one’s past. It could, however, be argued that the alteration of one’s past does not come at the expense of altering one’s values. Nevertheless, we argue that regardless of changes in one’s values, altering one’s past is a sufficient threat to the “true self” account of authenticity, as one’s narrative becomes altered disallowing the agent from seeing their life accurately, or in simpler terms, it is a threat to truthfulness/clear-headedness. In Erler’s account of “true self”, he writes “If she fails to remain true to herself when doing so would have been at least *prima facie* praiseworthy in her specific circumstances (again, if done for the right reasons), I will count her choice or action as inauthentic”. Therefore, MMTs are a threat to the “true self” account of authenticity regardless of its intrinsic value. Overall, while complex, it is clear that MMTs have a huge impact on authenticity and can lead to an inauthentic life. We will, however, point out that there are proponents of MMTs that would argue that MMTs can inversely aid in promoting authenticity through the relief of “invasive” memories that might hinder one from being oneself or the ideal self. However, with the definition of “true self” being the actual self rather than the ideal self, authenticity remains an issue. Similarly, patients with short-term memory dysfunctions such as early-stage dementia [48] could find that memory enhancement MMT benefits authenticity, as the restoration of short-term memory (leading to long-term memory) would lead to better clear-headedness/truthfulness, hence, a more authentic “true-self”. This does not, however, preclude the negative unwanted side effects that may occur with the use of MMTs, and their potential negative consequences on authenticity must also be considered. Regardless, for the purpose of this paper (practical applications), it would be more important to highlight the potential pitfalls rather than the benefits (which are mostly known) to allow the agent to make an informed decision.

### Ethical issues of MMTs

Development of MMTs have been progressing at a rapid pace, with new techniques and methodologies advancing ever closer to clinical applications. The use of such techniques raises new ethical issues and considerations that need to be addressed. In this section, we will highlight three ethical issues, namely unwanted consequences, guilt, and consent to change one’s values. We will attempt to analyse these issues using the frameworks discussed above. In these scenarios, one might argue that most MMTs are not irrevocable or irreversible, and hence any ethical issues faced can simply be resolved by discontinuing the treatment. We argue that while MMTs can be stopped, the changes to memory have long-lasting effects that are rather more permanent. For example, memory-erasing MTT is irreversible as we cannot reinstate the memory once we have removed it (compared to the inhibition of memory). Similarly, while memory enhancement MMT can be stopped, the “strong” memories already created by the memory enhancement MMTs will remain, including associated effects (e.g., anxiety or addiction) or other problems after discontinuing the treatment. The analysis of these effects, therefore, still holds value regardless of the permanence of the technology itself.

#### Unwanted consequences

Although the precision of MMTs is increasing, there is concern from unwanted consequences. For example, memory enhancement MMTs tend to overgeneralize the enhancement of memories, which has heavy implications on anxiety and addiction disorders. A person treated with memory enhancement MMTs could go through a traumatic event and develop strong overgeneralized fear memory, which in turn leads to anxiety disorder. Similarly, the use of memory enhancement MMTs in a person who is normally in control of their alcohol intake might lead to an overgeneralized enhancement of memory, which could mean a stronger association between alcohol and the brain’s reward circuitry leading to addiction. Most studies on memory editing MMTs have been done in animal models, but transitioning to humans comes with complex issues such as negative effects on declarative memory. For example, a soldier returning home from war might undergo memory editing MMT to treat PTSD to dull the fear response associated with traumatic memories. Besides disrupting fear memory, an unwanted and perhaps harmful side effect might be a disruption of the declarative memory of the time spent in the war. How then might these issues affect our ethical consideration of the use of MMTs? We argue that the use of MMTs is permissible if the outcome means an overall closer alignment to that of the Golden Mean. In this argument, we subscribe to the perfectionist view instead of the liberal view that the Golden Mean is one of the best possible virtues of life, rather than a best personal approach. This is supported by the neo-Aristotlean perspective, such as that of Julia Annas, in which we strive towards the impossible perfectionist model of the Golden Mean. Therefore, there is a need to consider the benefits of MMTs versus the potential unwanted consequences in the lens of what brings the agent closer to the “perfect self”. We acknowledge that an individual might not be able to identify the “best” outcome or understand the consequences fully before actually using MMTs, which is a whole other ethical consideration covered in the next section. Practically, ethical consideration means that in order for the use of MMTs to be permissible in regards to attaining the Golden Mean, it is crucial that we understand the possible outcomes and unwanted consequences, and that these are properly communicated to the patient. A bigger issue, however, is the ethical concerns of authenticity. Using MMTs may lead to an overgeneralized recall or off-target disruption of memories, which in turn can lead to changes in one’s values. What effect does this have on the three accounts of authenticity? In wholeheartedness, a change in one’s preference due to the use of MMT means that the agent no longer identifies with it wholeheartedly. However, one might argue that it is an unwanted consequence that changes the agent’s preference, yet they wholeheartedly identify with this change and thus it would not be inauthentic. In the existentialist account, the added issue of MMTs “controlling” one’s actions has implications on the agent’s ability to act honestly, as there would always be “blame” that could be placed on the unwanted consequences due to MMTs, hence, disowning one’s freedom. While one might argue that it is an unwanted consequence (be it a beneficial or harmful side effect), yet the agent does not intentionally deceive themselves and it would thus still be authentic. The direct action of using MMTS, however, could be seen as inauthentic, especially if the possibility of side effects is known. Although it might be harsh to charge a person with bad faith, in this case, we argue that in some situations of implicated inauthenticity, there is a degree of permissibility (this is discussed later in section 6). Lastly, unwanted consequences in terms of harmful side effects (i.e., developing anxiety or addiction issues) from the MMT that directly affect the “true self” account of authenticity might disallow the agent to remain true to themselves, even though the treatment might be intrinsically beneficial. Overall, we argue that although the ethical issues of unwanted consequences can be overcome by analysing them through the lens of the Golden Mean, its threat to authenticity remains an issue. We acknowledge, however, that as the research progresses and with an increased understanding of memory and how MMTs such as neuromodulation affect memory processes, we might be able to overcome these unwanted consequences, rendering these arguments moot. In the meantime, acknowledging such issues and their implications and understanding how to deal with them are still important.

#### Guilt

A major ethical issue is the use of MMTs to absolve guilt. A hypothetical scenario was presented by both Erler [9] and *Beyond Therapy* [47] in the *Lady Macbeth Case*. In this scenario, Lady Macbeth is guilt-stricken for her role in pushing her husband to murder King Duncan. To alleviate her guilt, she decides to use MMTs to alter her memories, which then raises the question as to whether it is ethically permissible for her to do so. Looking at it through the lens of Aristotle’s Nicomachean Ethics, it can seem like a permissible action. Quoting Aristotle:

“And of this nature Happiness is mostly thought to be, for this we choose always for its own sake, and never with a view to anything further” [.. .] “So then Happiness is manifestly something final and self-sufficient, being the end of all things which are and may be done.” (Nicomachean Ethics, Book 1, Chapter IV).

If MMTs indeed bring happiness (through lack of guilt) to Lady Macbeth, should she not then pursue it? As previously mentioned, Aristotle states we should “feel [emotions] when we ought, on what occasions, towards whom, why, and as, we should do”, suggesting the appropriateness of emotions needs to be considered. In this situation, we would argue that guilt is an appropriate emotion for Lady Macbeth’s actions, as it might prevent her from doing something similar in the future. However, the “happiness” achieved through MMTs in this situation should not contribute towards the Golden Mean, but rather creates an inappropriate emotion. This leads to the issue of a threat to authenticity, as pointed out by the President’s Council on Bioethics (2003), in that the use of MMTs “however desirable to alleviate guilty or painful consciousness, could subtly reshape who we are”. Interestingly, there is a case to be made that Lady Macbeth’s actions were authentic in terms of wholeheartedness. In this scenario, Lady Macbeth is acting upon her preferences (that is to not feel guilty) and she wholeheartedly believes/identifies with her authentic self as one without guilt. However, in the existentialist account of authenticity, by using MMTs, Lady Macbeth is engaging in self-deception and hence can no longer be justified in making an authentic choice. Lastly, in the “true self” account, Lady Macbeth uses MMTs to alter her narrative, deliberately falsifying it to absolve her guilt, and therefore, no longer leads an authentic life. Overall, it would appear that the use of MMTs to alleviate guilt has issues with both the Golden Mean and authenticity. Conversely, what about unnecessary guilt such as parents’ guilt due to the accidental death of a child. The parents would feel guilty for not being able to protect their child, even though this was through no fault of their own. In such a case, would it be permissible to use MMTs to alleviate guilt? The difference between this case and that of Lady Macbeth is that the feelings of guilt in the parents are not appropriate, and thus the pursuit of happiness in alleviating the guilt is valid towards the Golden Mean. However, the use of MMTs would still lead to a form of self-deception by altering one’s narrative, posing a threat to authenticity. We do acknowledge that this is contentious in that the agent would acknowledge the use of MMT and its effects, and hence, would not be deceiving themselves. However, we argue that forceful alteration of a narrative can indeed be inherently self-deceiving. Regardless, the threat of authenticity is valid in either case.

#### Consent and change in one’s values

Several fundamental questions emerge from the use of MMTs: Is there a change in one’s values after the use of MMTs? How does this affect user consent? Is there a difference in agency before and after use? For example, we present a scenario in which a patient suffering from an anxiety disorder such as PTSD decides to use a memory editing MMT to erase the underlying fear memory with full understanding that this will also remove the vivid nature of the memory. The patient might agree to this due to the invasive nature of PTSD, but might subsequently regret it when they no longer suffer from PTSD after the MMT treatment. Could a patient with an intrusive fear memory properly give consent to alter said memory? Is the pre-MMT or post-MMT self considered the “true self”?

We present another scenario in which a person, fully understanding the possible side effects, decides to use memory enhancement MMTs to boost their productivity, but post-MMT develops anxiety and low self-esteem due to the inability to forget small shortcomings, subsequently leading to regret over the procedure. Could that person fully understand the possible side effects without having gone through them? If the post-MMT self is different from the pre-MMT self, to which “self” do we attribute agency and value in the use of the MMT. How do we reconcile the difference in values of the pre- and post-MMT self? In this case, we argue firstly that the pre- and post-MMT self are equally authentic, but are now different selves. However, assuming that the decision made by the pre-MMT self was informed and autonomous, we argue that we have to look at this situation through the lens of the pre-MMT self rather than that of the post-MMT self. An analogy would be a chess player, who after making a move, realises it will lead to losing the game. The post-decision self might regret the decision given that they now have new knowledge about how it affects the game. However, the pre-decision self made the decision authentically based on what the pre-decision self thought was the best move to win in that scenario. The “new knowledge” of the post-decision self, therefore, does not preclude the responsibility of the consequences of the decision by the pre-decision self. In which case, the responsibility of making an authentic and good decision fully lies with the pre-decision self and their knowledge at that time. Similarly, the ethical responsibility and the decision made lies with the pre-MMT self rather than with the post-MMT self. The post-MMT self then has to take responsibility for the consequences of the pre-MMT self and to live authentically with the new “true self” post-MMT. This argument, however, does not address the more difficult issue of whether a patient with a mental disorder can indeed truly consent to use MMTs. According to the Mental Health Ordinance in Hong Kong (where the authors reside), Cap136 (1997):

“A mentally incapacitated person is incapable of giving such consent if that person is incapable of understanding the general nature and effect of the treatment or special treatment.”

For certain situations in which a patient has severe psychosis or disability that prevents them from even remotely understanding the treatment, it is clear that the decision would be made for them. However, in the two scenarios mentioned above, the individuals are not entirely debilitated and can still understand the general nature and effects of MMTs. When using MMTs to enhance productivity, assuming the person is mentally healthy pre-MMT, there is no issue with consent (we argued it should be looked at through the lens of the pre-MMT self). However, would a patient with PTSD be able to understand sufficiently the general nature of the treatment given the debilitating anxiety? It could be argued that the intrusive nature of the fear memory debilitates the patient from truly understanding the nature and effects of using memory-erasing MMT. The question is where to draw the line in patients with mental disorders who can and cannot give consent. Although there are guidelines that clinicians follow, the ethical quandary of this question remains. In such cases, a practical application of the Aristotle’s Golden Mean could be useful. Assuming that the pre-MMT self makes an authentic decision with knowledge of the possible consequences, then the Golden Mean through the lens of the pre-MMT self would help to make such a decision. We make a distinction here between knowledge and understanding, with the former being a patient with the mental capacity to understand the consequences, and the latter being a patient who fully understands the implications and the content of the consequences. For example, if a patient with PTSD considers erasing a significant but intrusive memory that has a major role in the patient’s identity, there are two things we need to think to about: 1) does the patient have the knowledge of the possible consequences (this includes both having the capacity to understand the content of the consequences and the availability of this knowledge), and 2) does erasure of the memory bring the patient overall closer to the Golden Mean. We do acknowledge that there would still be issues with authenticity, but in this case, the use of the Golden Mean could be a step to address the issue of consent, and more thought can then be given to the implications on authenticity. Nevertheless, if the two above criteria apply, we would argue that regardless of the post-MMT self’s preference on that decision, the pre-MMT self’s decision was still valid and it is the responsibility of the post-MMT self to live with this decision.

### Practical applications of ethical theories

In the previous sections, the ethical implications of using MMT were considered according to two major philosophical frameworks, Aristotle’s Golden Mean and authenticity. In this section, we propose a practical framework to aid in the ethical considerations of the decision process for the use of MMTs.

#### The model

**Fig. 1:**
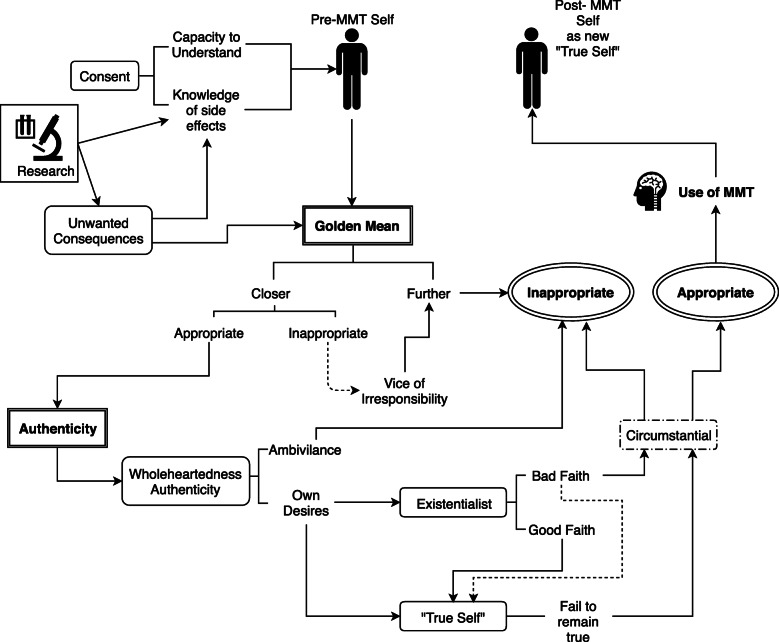
A systematic model for the practical analysis of the decision to use MMTs. The flowchart combines the ethical theories of Aristotle’s Golden Mean and authenticity in an attempt to systematically guide one’s thought processes in deciding the appropriateness of using MMTs in a given situation. Figure and clipart in it was drawn using an opensource diagramming software (diagrams.net)

The proposed practical model aims to systematically use the theoretical frameworks discussed above in the process of deciding whether MMTs are appropriate in a given circumstance. This model is not designed to dictate or justify a decision per se, rather the user is systematically guided through a list of ethical considerations to decide on the appropriateness of using MMTs. Figure 1 illustrates the proposed framework as a flow chart that consolidates the two ethical frameworks with their related ethical issues as discussed above. We believe this framework can aid in physicians’ discussions with patients on the appropriateness of use of MMTs. Although the use of the Golden Mean lies more on the physicians’ side, whereas the final decision on the use of MMT depends on the patients’ assessment of the effects on their authenticity, considerations from both sides on both issues are warranted.

In this model, the frameworks are applied to the pre-MMT self. In the first layer, consent is a prerequisite for considering the use of MMTs, encompassing both the capacity to understand and knowledge of the side effects that are, at least in part, caused by unwanted consequences. Advances in neuroscience research play a crucial part in the consent to use MMT. With increased knowledge and further advances in MMTs research, particularly in the precision of these methods, these unwanted consequences are reduced, and we foresee these factors will become less of a concern. As part of the first layer, we take into consideration the unwanted consequences of using MMT through the lens of the Golden Mean, which essentially has two outcomes, closer or further from the Golden Mean. Further from the Golden Mean clearly indicates it would be inappropriate to use MMT, whereas closer to the Golden Mean would require further consideration of the appropriateness according to “feel them [emotions] when we ought” (as discussed in section 2.2). For example, in the *Lady Macbeth case* mentioned in section 4.2, using MMT would lead to a vice of irresponsibility, which is ultimately considered to be further away from the Golden Mean and thus inappropriate.

If it is appropriate in terms of the Golden Mean, then the next step is to look at the effects of MMT on authenticity. We argue that being authentic in the wholehearted sense (or “Frankfurtian” authenticity sense) is necessary to be ethically appropriate, regardless of the circumstances of MMT use. If a decision is made ambivalently and not out of the agent’s own desires, then it becomes fundamentally and ethically inappropriate, as it either indicates loss of one’s agency or is done without proper consideration (i.e., one must actively want to undergo MMT instead of passively accepting it or being made to). If it is truly out of the agent’s own desire, then we need to further consider the decision using the two other forms of authenticity. We need to consider the existentialist account of authenticity to understand if the decision is made in good faith or bad faith. If the decision is made in bad faith, we do not think it immediately excludes the use of MMT, but rather this becomes another consideration for its use. Quoting Erler [9]:

“Nevertheless, the demands of authenticity need not always override any competing considerations. E.g., if his traumatic memories of abuse were causing him a lot of suffering that neither psychotherapy nor propranolol could be expected to sufficiently alleviate, erasing the memories might be morally permissible”.

We agree with this statement in that we need to weigh the benefits of MMT with the demands of authenticity, although we should not altogether ignore its implications on authenticity. Regardless of whether the decision is made in good faith or bad faith, we also need to take into the “true self” account of authenticity. In this case, we agree with Erler that the use of MMTs in all cases leads to living inauthentically (hence the single outcome in the figure). Similarly, we do not think this precludes the use of MMTs, rather it is also another consideration for its use. We, therefore, argue that the appropriateness of using MMTs depends on the circumstance. This “anti-climactic” conclusion might perhaps show a failure in the aim of this flowchart; however, this chart is meant as a systematic guide to one’s thought processes through the ethical considerations. Nevertheless, if considered appropriate and the agent decides to undergo the MMT, as argued in section 4.2, the post-MMT self is equally as authentic as the pre-MMT self and is responsible for living with the pre-MMT self’s decision. We, therefore, argue that the post-MMT self then becomes the new “true self” and subsequent considerations should be made as the post-MMT self.

### Caveats and considerations

#### Inauthenticity can be permissible

As stated above, we do not think that inauthenticity in the existentialist or “true self” accounts immediately excludes the use of MMT. In the existentialist accounts, we argued above that the agent disowning their freedom through blaming their actions on the use of MMTs and through self-deception in the use of MMTs are threats to authenticity. Nevertheless, we admit it would be harsh to charge one with bad faith for those willing to risk the harmful side effects of MMTs for the potential good that it might produce. Furthermore, personal responsibilities challenge Sartre’s view of freedom, in that we have responsibilities in our lives that perhaps realistically limit our freedom. Although an existentialist might argue that this constitutes bad faith as said responsibilities can be seen as “societal pressures”, it realistically remains a consideration in how we act. We, therefore, argue that there are situations in which certain inauthenticities can be moral, in that certain “inauthenticities” can lead to a situation where the agent “feels them [emotions] when we ought”. In a scenario called “Carl’s case” proposed by Erler [9], due to abuse, Carl has ended up in a life of crime. He is offered memory-erasing treatment to erase the traumatic experiences that turned him into a hardened criminal in exchange for parole. If Carl accepts this deal, it could be seen as inauthentic, as he chooses to deceive himself through memory erasure due to “societal pressures”. However, in such a case, we would argue that as long as he chooses this not out of ambivalence, but by his own desires (assuming that his own desires are to integrate back into society rather than an easier alternative to time in prison), then the inauthenticity in the existentialist account is morally permissible as the benefits outweigh the loss of authenticity.

Given that we have argued that all uses of MMTs would be inauthentic in the “true self” account of authenticity, we similarly argue that in such circumstances where the outcome outweighs the loss of authenticity, then the use of MMT is permissible. In “Carl’s case”, the use of MMT to erase memories would be considered inauthentic in the “true self” account as it entails changing the past narrative of Carl’s life, and therefore, he is no longer living truthfully. However, given such a circumstance, we would argue the use of MMT is permissible. Furthermore, one can argue that none of our memories are fully real (at least episodic memories), but rather a unique personal perspective on what has occurred. Shaw and Porter [38] showed that full episodic memories can be implanted in over 70% of subjects through suggestive memory retrieval techniques. Although this research focused on the criminal psychology of false confessions, it has huge implications on our memories and suggests that our memories are incredibly malleable and likely not fully accurate. In this case, one can argue that authenticity in the “true self” account is impossible to fully achieve (at least if we look at it as “truthfulness”), but rather we should look at it as an attempt to be reasonably accurate instead of “photographically” accurate. We, therefore, argue that the “true self” account of authenticity, while useful in examining the authenticity of an action, should not immediately exclude the use of MMTs, but rather aid in the decision-making process by systematically allowing the potential user to consider the implications according to their own narrative. Overall, a more useful approach would be to use *pro-tanto* reasoning. For example, the treatment of psychiatric disorders overrides inauthenticity. Quoting Savulescu and Sandberg’s discussions on “love drugs” [49]:

“Even if love were not authentic, authenticity is not an overriding or exclusive value. People can trade a degree of authenticity for other values in their lives.”

#### Altering implicit memories

The use of memory editing/erasing MMTs have tended to focus on altering implicit memories instead of explicit ones. For example, memory-erasing MMTs are used to treat anxiety disorders by removing the “fear element” from the memory rather than to erase the whole memory itself. This brings up the question of whether altering implicit memories still counts as self-deception (and hence inauthentic) if one is aware that the MMT is reducing the emotional component attached to the explicit memory? This has implications for both the existentialist and “true self” accounts of authenticity. Self-deception in the existentialist’s account would be to deny the responsibility of freedom. In this case, altering implicit memories could arguably involve no self-deception as the agent acknowledges the use of MMTs and exercises their freedom to do so. However, it should be noted that for the agent to remain authentic, the subsequent choices influenced by changes in implicit memory after using MMT should not be blamed on the MMT itself. The “true self” account of authenticity, however, is arguably “less forgiving” on the matter. Changes in implicit memory, regardless of the acknowledgement, can be considered as not remaining true to oneself as it alters the narrative of one’s past, even if it is only the emotional component. While it can be argued that changes in implicit memory, if acknowledged, can still be authentic to the “true self” as it involves no self-deception in the use of MMT or the effects on the present self, there remain issues of “clear-headedness” given that one’s narrative is altered. One can make the argument that “clear-headedness” can still be achieved if the agent acknowledges the use of MMT and understands its effects. However, we argue that by altering memory in any sense, even implicit memory, one is only partially truthful and no longer fully truthful, and therefore, “clear-headedness” concerns still remain, which in this paper we have grouped with “true self” for simplicity. Again, we do not think this excludes the use of MMTs, but rather provides another important consideration. We, therefore, argue that if one acknowledges and embraces the changes caused by MMT, the use of MMT then becomes part of one’s narrative and can perhaps be authentic in that sense. In both situations, there is a possibility of self-deception even if the MMT exerts its effects only on implicit memories, but we argue a way to ensure authenticity is to fully embrace the post-MMT self as the new “true self”, which we have previously discussed. In this case, the post-MMT self would not place the responsibility of freedom on the use of MMT as the post-MMT self is now accepted as the new/present “true self”. Embracing the post-MMT self would also mean embracing the use of MMT and the consequences, incorporating them into one’s narrative and thus remaining authentic (at least in the “true self” account). Overall, we argue that while changes in implicit memories can indeed lead to self-deception, embracing the post-MMT self would resolve most of these issues.

#### Conflicts in the Golden mean

An issue in the use of the Golden Mean is that developing one aspect of the Golden mean could conflict with other aspects. For example, the use of memory enhancement MMT might be able to reverse memory impairments bringing that aspect towards a Golden mean, but at the same time, fear and anxiety due to increased memory recall creates excess fear that pulls these aspects away from the Golden mean. In such cases, we can take the rigorist view of the Stoics in that none of us are perfectly virtuous, except through the imagination of “the ideal sage”. However, unlike the stoics, we subscribe to the view that we can still be virtuous at a “learner” level, according to Julia Annas’ description in her book “Intelligent Virtue” [50]:

“The learner can still properly be called virtuous; apologizing for wrongdoing is doing the right thing only in the sense that it is acceptable, minimally OK, better than not apologizing, and this is precisely appropriate for the stage of the learner. It would be problematic to say a truly virtuous person could be in this position since the truly virtuous person has the understanding not to be in the situation in the first place.”

Taking this into consideration, how then can we as learners strive towards the virtuosity of “the ideal sage”, and how does this relate back to the question at hand? We argue that the perfect Aristotelian virtue is impossible (as thought by the Stoics), and it is permissible for us as learners to use MMT to improve one aspect over another if the overall benefits outweigh the sacrifices to the other aspects, such that the overall benefits brings the agent closer to the Stoic’s perfectionist view of virtue and the Golden Mean. We do, however, further argue that the “sacrificed” aspect should still be at least barely acceptable, and by barely acceptable, we mean in the sense of what Annas writes on ‘right’ being ‘barely acceptable’ [50]:

“We say both, ‘He acted tactlessly and blunderingly, but at least he did the right thing,’ (referring to barely acceptable) and ‘He did the right thing in responding to the situation in an exemplary way.’”

Although Annas states that “‘right’ is a ‘thin’ ethical concept, lacking independent ethical content of its own, as opposed to ‘thick’ ethical concepts like virtues”, she also writes that “‘right’ is adjusted to the developmental account of virtue” and that it “can range from what the learner does to what the truly virtuous person does” [50]. We, therefore, argue we are all, in one form or another, learners in virtue, and ‘right’ is a useful concept that should be considered when using MMTs. Nevertheless, the more difficult quandary is perhaps one of practicality, as it is virtually impossible to quantify each aspect of virtue, much less quantify the changes due to MMT. Perhaps then it comes down to what the agent prioritizes, their understanding of the consequences, and whether they accept the post-MMT self as the new “true self”, and from this point on to continue as a learner striving towards virtue.

## Conclusions

The development of MMTs have been progressing at a rapid rate, advances in techniques such as neuromodulation bring us closer to being able to effectively modulate memories. The implications of this are tremendous and must be handled carefully [6, 7]. Although MMTs have immense power in treating debilitating disorders such as dementia and anxiety, there are many ethical implications that must be considered depending on the circumstance. In this paper, we highlighted two major ethical areas for consideration in the use of MMTs, namely Aristotle’s Golden Mean and authenticity. We then provided a practical model for systematically navigating the ethical issues in the decision to use MMTs. Lastly, we highlighted certain considerations in the use of our proposed framework. At face value, it might appear that the framework puts severe limitations on the use of MMTs, as a large proportion of flow chart ends with “no” to the use of MMTs, but this is not an indictment against the use of MMTs, but rather a guide for the agent to consider the implications before making a decision. Most importantly, we argue that the agent must accept the post-MMT self as the new “true self” regardless of the outcome. While the reversibility of many MMTs (by discontinuing treatment) could mean that a patient could cease treatment if they do not endorse it as constituting oneself, the effects of permanence of the altered, erased, or obtained memories still contribute to what we consider as the new “true self”, which we argue must be accepted by the agent. Perhaps the more crucial point would be that research into how MMTs actually function, their harmful side effects/unwanted consequences, and their beneficial outcomes would facilitate the ethical considerations involved in the use of MMTs. Regardless, the developmental trajectory of MMTs seems to suggest that clinic applications are getting closer. Hence, we need to be prepared to deal with the ethical implications and to develop the means for both clinicians and agents to understand the implications of the use of MMTs. Lastly, these ethical concerns need to be discussed in a wide range of disciplines to develop the best strategies and policies to deal with MMTs. While this paper is limited in its engagement with neuroethics literature on the topic in the interest of focus and due to limitation of space, other authors have previously discussed ethical considerations on memory manipulation in-depth and can be referred to for further reading on the topic [51, 52]. Other ethical concerns on the use of MMT including consent [53, 54], identity [55], and managing expectations [56] have also been previously discussed, and integrating them into the current framework would be highly important for its practical application. Similarly, concerns on equity in the use of MMTs and the effects of MMTs on society [57] must be considered beyond the issues presented in this paper. If all this can be achieved, we believe the ethical use of MMTs is not only possible, but would be of tremendous benefit to many people suffering from memory-related mental disorders.

## Data Availability

Not Applicable.

## Abbreviations

DBS: Deep Brain Stimulation
LTP: Long-Term Potentiation
MMTs: Memory Modulating Technologies
PTSD: Post-Traumatic Stress Disorder
